# Reliable differentiation of *Meyerozyma guilliermondii* from *Meyerozyma caribbica* by internal transcribed spacer restriction fingerprinting

**DOI:** 10.1186/1471-2180-14-52

**Published:** 2014-02-28

**Authors:** Wahengbam Romi, Santosh Keisam, Giasuddin Ahmed, Kumaraswamy Jeyaram

**Affiliations:** 1 Microbial Resources Division, Institute of Bioresources and Sustainable Development (IBSD), Takyelpat Institutional Area, Imphal 795001, Manipur, India; 2 Department of Biotechnology, Gauhati University, Guwahati 781014, Assam, India

**Keywords:** *Meyerozyma guilliermondii*, Emerging infectious yeasts, ITS-RFLP, mtDNA-RFLP, PFGE karyotyping, *Soibum*

## Abstract

**Background:**

*Meyerozyma guilliermondii* (anamorph *Candida guilliermondii*) and *Meyerozyma caribbica* (anamorph *Candida fermentati*) are closely related species of the genetically heterogenous *M. guilliermondii* complex. Conventional phenotypic methods frequently misidentify the species within this complex and also with other species of the *Saccharomycotina* CTG clade. Even the long-established sequencing of large subunit (LSU) rRNA gene remains ambiguous. We also faced similar problem during identification of yeast isolates of *M. guilliermondii* complex from indigenous bamboo shoot fermentation in North East India. There is a need for development of reliable and accurate identification methods for these closely related species because of their increasing importance as emerging infectious yeasts and associated biotechnological attributes.

**Results:**

We targeted the highly variable internal transcribed spacer (ITS) region (ITS1-5.8S-ITS2) and identified seven restriction enzymes through *in silico* analysis for differentiating *M. guilliermondii* from *M. caribbica*. Fifty five isolates of *M. guilliermondii* complex which could not be delineated into species-specific taxonomic ranks by API 20 C AUX and LSU rRNA gene D1/D2 sequencing were subjected to ITS-restriction fragment length polymorphism (ITS-RFLP) analysis. *Taq*I ITS-RFLP distinctly differentiated the isolates into *M. guilliermondii* (47 isolates) and *M. caribbica* (08 isolates) with reproducible species-specific patterns similar to the *in silico* prediction. The reliability of this method was validated by ITS1-5.8S-ITS2 sequencing, mitochondrial DNA RFLP and electrophoretic karyotyping.

**Conclusions:**

We herein described a reliable ITS-RFLP method for distinct differentiation of frequently misidentified *M. guilliermondii* from *M. caribbica*. Even though *in silico* analysis differentiated other closely related species of *M. guilliermondii* complex from the above two species, it is yet to be confirmed by *in vitro* analysis using reference strains. This method can be used as a reliable tool for rapid and accurate identification of closely related species of *M. guilliermondii* complex and for differentiating emerging infectious yeasts of the *Saccharomycotina* CTG clade.

## Background

*Meyerozyma guilliermondii* is a genetically heterogenous complex belonging to the *Saccharomycotina* CTG clade [1]. This complex consists of phenotypically indistinguishable and closely related species namely *Meyerozyma guilliermondii* (anamorph *Candida guilliermondii*), *Meyerozyma caribbica* (anamorph *Candida fermentati*), *Candida carpophila*, *Candida smithsonii*, *Candida athensensis*, *Candida elateridarum* and *Candida glucosophila*[2-6]. Apart from its presence in healthy human [7,8], *M. guilliermondii* also exists in clinical [3,9] and environmental samples [10]. This organism is widely studied in various aspects due to its clinical importance, biotechnological applications and biological control potential [11].

*C. guilliermondii* is regarded as an emerging infectious yeast of the non-albicans *Candida* (NAC) species group which accounts for 1 – 5% of nosocomial blood stream infections worldwide [9,12,13]. However, in certain geographical regions such as Brazil, India and Italy, over 10% of all the candidaemia cases are caused by this species [14]. The threat posed by this organism is ever increasing due to the decreased susceptibility and emergence of strains resistant to antifungal drugs like polyene (amphotericin B) and azoles (fluconazole and itraconazole), leading to mortality in candidaemia patients [9,12,15]. *C. fermentati* has been rarely found to be associated with candidaemia [16,17]. But due to the poor discernability of *C. fermentati* from *C. guilliermondii*, they are commonly misidentified in clinical laboratories. Apart from being organisms of clinical importance, *M. guilliermondii* and *M. caribbica* are often linked with fermented foods [18-20]. *M. guilliermondii* is known for the production of flavour compounds in fermented food products [21]. Further, in a study with soybean paste fermentation*, M. guilliermondii* and *M. caribbica* have been claimed for the efficient production of isoflavone aglycone which is a widely known bioactive compound for its various health promoting functions [22]. *M. guilliermondii* is a flavinogenic yeast which is known for the overproduction of vitamin B_2_ (riboflavin) [23]. Moreover, isolates of *M. guilliermondii* and *M. caribbica* have exhibited great potential in the biological control of fungi responsible for postharvest spoilage of fruits and vegetables [24-26]. These yeast species with enhanced biological control efficacy have emerged as a potential alternative to the conventional fungicide treatment. Considering the various importance and applications of the two species, there is a need for the development of accurate and reliable method to identify and distinctly discriminate the closely related species.

Current methods of yeast identification, mostly in clinical practice, are mainly based on the conventional and rapidly evolving commercial phenotypic and biochemical methods. However, such methods are often unreliable for accurate identification of closely related yeast species [13,27]. According to recent studies, *M. guilliermondii* and *M. caribbica* are extremely difficult to differentiate by the phenotypic methods [28-31]. We also faced similar problem during differentiation of yeast isolates from *soibum*, an indigenous fermented bamboo shoot product of North East India (Additional file 1: Table S1). The widely used API 20 C AUX yeast identification system and sequencing of large subunit (LSU) rRNA gene D1/D2 domain failed to give proper species-level taxonomic assignment to these isolates (Additional file 1: Tables S2 and S3). Moreover, the phylogenetic tree reconstructed from the publicly available D1/D2 sequences of different strains of *M. guilliermondii* and *M. caribbica* failed to discriminate the two species (Additional file 2: Figure S1).

Several attempts have been made using molecular approaches such as DNA base composition, electrophoretic karyotyping [6,32], multi locus sequence typing (MLST) [3], multi locus enzyme electrophoresis (MLEE), randomly amplified polymorphic DNA (RAPD) [4], sequencing of internal transcribed spacer (ITS) [28,30], intergenic spacer restriction fragment length polymorphism (IGS-RFLP) [29] and RFLP of housekeeping genes such as riboflavin synthetase gene *RIBO*[17] in order to resolve the misidentification. Some recent studies have claimed that the matrix-assisted laser desorption ionization-time of flight mass spectrometry (MALDI-TOF-MS) is advantageous over previous approaches for reliable identification of clinically important NAC and non-*Candida* yeast species [28,31,33,34]. Unfortunately, MALDI-TOF-MS requires reference spectra of accurately identified closely related strains otherwise the results may be erroneous. On the other hand, the sequence-based studies have considered the ITS1-5.8S-ITS2 region as universal DNA barcode for yeast identification [35] and the RFLP of ITS1-5.8S-ITS2 region has successfully separated the closely related species in the genera *Candida* and *Pichia*[36,37]. Therefore, in this study, we targeted the ITS1-5.8S-ITS2 region to develop a simple RFLP method for accurate taxonomic assignment of *M. guilliermondii* and *M. caribbica*.

With this background, the aim of the present study was (i) to perform *in silico* prediction of restriction enzymes to discriminate *M. guilliermondii* and *M. caribbica* using the publicly available ITS1-5.8S-ITS2 sequences, (ii) to evaluate the selected enzymes by *in vitro* ITS-RFLP analysis of ambiguously identified 55 yeast isolates for species-specific taxonomic assignment, and (iii) to validate the taxonomic assignment by ITS1-5.8S-ITS2 sequencing, mitochondrial DNA (mtDNA)-RFLP and pulsed field gel electrophoresis (PFGE) karyotyping.

## Methods

### Yeast isolates and strains

The yeast isolates used in the present study are listed in Additional file 1: Table S1. These isolates were obtained from samples collected at different stages of indigenous bamboo shoot fermentation for the production of *soibum* in Manipur state of North East India [38]. The sample (10 g) was homogenized in 90 mL of sterile physiological saline (1 g/L bacteriological peptone, 8.5 g/L NaCl, pH 6.1) using Stomacher® 400 Circulator (Seward, Worthing, West Sussex) at 250 rpm for 3 min. The yeasts were isolated by serial dilution spread-plating of the above homogenate on yeast extract peptone dextrose (YEPD) agar medium (pH 6.5) (HiMedia, Mumbai, India) containing 100 μg/mL each of filter-sterilized ampicillin and tetracycline (Sigma-Aldrich, Bangalore, India), followed by incubation at 30°C for 48 − 72 h under aerobic conditions. All the isolates were purified by sub-culturing twice on the same agar medium and preserved at −80°C in YEPD broth containing 10% (v/v) sterile glycerol (Sigma-Aldrich). For short term storage, the cultures were maintained at 4°C on YEPD agar. The type strain *C. guilliermondii* ATCC 6260 used for comparison was obtained from American Type Culture Collection.

### Phenotypic characterization and morphological observation

Phenotypic identification of the yeast isolates was carried out using the API 20 C AUX yeast identification system (bioMérieux, New Delhi, India) following manufacturer’s instructions. Colony and cell morphology of the isolates were studied using SZ-PT stereo binocular microscope (Olympus, Japan) and BX61 phase contrast microscope (Olympus).

### *In silico* analysis and restriction enzyme selection

The full length ITS1-5.8S-ITS2 sequences of *M. guilliermondii* and *M. caribbica* were retrieved from NCBI (http://www.ncbi.nlm.nih.gov/) and Centraalbureau voor Schimmelcultures (CBS-KNAW) yeast nucleotide databases (http://www.cbs.knaw.nl/Collections/Biolomics.aspx?Table = CBS + strain + database). Type strain sequences of the two species, *C. guilliermondii* ATCC 6260 [GenBank: AY939792.1] and *M. caribbica* CBS 9966 (http://www.cbs.knaw.nl/Collections/BioloMICS.aspx?Link = T&TargetKey = 14682616000000137&Rec = 36291&Revert = F) were subjected to *in silico* PCR amplification using primers ITS1 (5′-TCCGTAGGTGAACCTGCGG-3′) and ITS4 (5′-TCCTCCGCTTATTGATATGC-3′) [39] to trim off the untargeted regions on both 5′ and 3′ ends of the sequences using the online Sequence Manipulation Suite (http://www.bioinformatics.org/sms2/pcr_products). Using NEBcutter, version 2.0 (http://tools.neb.com/NEBcutter2/), the ITS amplicon sequences were subjected to *in silico* restriction digestion using the commercially available type-II restriction endonucleases listed in the REBASE database (http://rebase.neb.com/) [40] to select the enzymes which cut the two sequences differently at not more than 5 cleavage sites. Multiple sequence alignment of 10 additional ITS1-5.8S-ITS2 sequences of different strains from different ecological niches for each species was performed using Clustal X, version 2.0 (http://www.clustal.org/clustal2/) and BioEdit, version 7.2.0 (http://www.mbio.ncsu.edu/bioedit/bioedit.html) to confirm the taxa-specificity of the selected restriction enzymes.

### DNA extraction

DNA was extracted from pure cultures as cell-free DNA lysate using lyticase-heat lysis method. Briefly, a single colony of 24 − 48 h old culture from YEPD agar was inoculated to 5 mL of YEPD broth supplemented with antibiotics, and incubated for 18 h at 30°C with shaking at 200 rpm. Cells were harvested from 1 mL of the culture broth at 5,000 g for 5 min (FA-45-24-11, Centrifuge 5424, Eppendorf, Hamburg, Germany). The cell pellet was washed twice with 1 mL sterile 0.5 M NaCl followed by sterile deionized water (Milli Q, Millipore, Molsheim, France). The cells were finally resuspended in 500 μL of 1× TE buffer (10 mM Tris-Cl, 1 mM EDTA, pH 8.0) containing 10 μL of lyticase (5U/μL) (Sigma-Aldrich) and incubated at 37°C for 1 h. After the incubation, the spheroplasts were lysed by heating at 95°C for 20 min. The crude cell-free lysate was collected by centrifugation at 10,000 g for 10 min at 4°C and the DNA was quantified spectrophotometrically (Nanodrop ND-1000, NanoDrop Technologies, Inc., Rockland, USA). The cell-free lysate with absorbance ratio (A_260/280_) of 1.8 − 2.2 was used for PCR analysis and stored at −20°C until required.

### ITS-RFLP

ITS1-5.8S-ITS2 was amplified from the cell-free DNA lysate using primers ITS1 and ITS4 mentioned elsewhere. The amplification was carried out in a 25 μL final reaction volume containing 50 ng of the genomic DNA as previously described [41]. The amplified ITS fragment was analyzed by 2.0% (w/v) agarose gel electrophoresis at 80 V in 0.5× TBE (45 mM Tris-borate, 1 mM EDTA, pH 8.0) buffer to check its intactness and absence of non-specific amplification. The PCR product (4 μL) was digested with 5 U of *Taq*I (Promega, Madison, USA) in a 10 μL reaction volume at 65°C as per manufacturer’s instructions. The restriction patterns were analyzed by electrophoresis of the 10 μL reaction volume on 2.0% (w/v) agarose gel in parallel with PCR 100 bp Low DNA ladder (Sigma-Aldrich) as molecular size standard. The electrophoresis was run at 80 V for 2 h in 0.5× TBE buffer. The gel was then stained in 0.5 μg/mL ethidium bromide solution for 30 min with rocking at 15 rpm on a platform rocker (Tarsons, Kolkata, India). After destaining for 30 min in sterile deionized water, the gel was photographed using ChemiDoc MP gel documentation system (Bio Rad, Hercules, USA). The restriction fingerprints were analysed for the absence or presence of discriminating fragments using GelCompar II software, version 6.5 (Applied Maths, Sint-Martens-Latem, Belgium).

### mtDNA-RFLP

A single colony of 24 − 48 h old culture from YEPD agar was inoculated to 5 mL of YEPD broth supplemented with antibiotics, and incubated for 18 h at 30°C with shaking at 200 rpm. The grown culture was inoculated into 50 mL of fresh YEPD broth (initial OD_600_ = 0.1) and incubated in the above conditions till mid-logarithmic growth phase (final OD_600_ = 0.4 − 0.8). Cells of 20 OD_600_ were harvested at 1,800 g for 5 min at 4°C (A-4-81, Centrifuge 5810R, Eppendorf). The mtDNA was extracted as previously described [42] with some modifications. The cells were resuspended and washed with 5 mL of yeast resuspension buffer (50 mM Tris-Cl, 20 mM EDTA, pH 8.0) and stored at −20°C for 10 min. Lyticase (50 U) (Sigma-Aldrich) was used to produce spheroplast and 15 μL of 1 mg/mL RNase A solution (Sigma-Aldrich) was added during cell lysis. The total DNA was precipitated at −20°C for 1 h. After quantifying the DNA content spectrophotometrically, the DNA was freeze dried, re-dissolved in sterile deionized water to a final concentration of 1 μg/μL and stored at −20°C till further use. Restriction digestion was carried out on 10 μg of the DNA in a 20 μL reaction volume using 10 U each of *Hae*III and *Hinf*I (Promega) according to manufacturer’s instructions. The restriction patterns were generated by 1.0% (w/v) agarose gel electrophoresis of the 20 μL reaction volume at 80 V in 0.5× TBE buffer for 4 h in parallel with 1 kb DNA ladder (Promega). After staining and documentation, the restriction fingerprints were subjected to cluster analysis using unweighted pair group method with arithmetic mean (UPGMA) algorithm on Jaccard similarity coefficients using GelCompar II. Composite data set of the restriction digestion profiles was generated with 1.0% position tolerance to generate the clustering. Bootstrap analysis with 1,000 replicates was performed to indicate the branch quality.

### Electrophoretic karyotyping

Intact chromosomal DNA for electrophoretic karyotyping using PFGE was prepared as previously described [32]. The electrophoresis was carried out in 1.0% (w/v) PFGE-grade agarose gel (Sigma-Aldrich) and 0.5× TBE buffer at 13 − 14°C and 150 V in contour-clamped homogeneous electric field electrophoresis apparatus (Gene Navigator, Amersham Biosciences, Uppsala, Sweden). The gel was run for 22 h with a switch interval of 90 s for 8 h followed by 105 s for 6 h and finally 120 s for 8 h in parallel with PFGE marker (225 − 22,000 kb) from *Saccharomyces cerevisiae* strain YPH80 (Sigma-Aldrich). Staining and documentation were performed as mentioned elsewhere.

### ITS and D1/D2 sequencing and sequence analysis

The representative isolates from each ITS-RFLP genotype group were randomly selected for sequencing ITS1-5.8S-ITS2 and LSU rRNA gene D1/D2 domain. ITS1-5.8S-ITS2 was PCR amplified as mentioned elsewhere. Amplification of D1/D2 region was carried out using primers NL1 (5′-GCATATCAATAAGCGGAGGAAAAG-3′) and NL4 (5′-GGTCCGTGTTTCAAGACGG-3′) as previously described [43]. The amplified products were purified using NucleoSpin® Extract II gel extraction kit (Machery-Nagel, Düren, Germany) following manufacturer’s instructions. The PCR products were sequenced using ABI 3100 Genetic Analyser (Merck, Bangalore, India) with the same primers used for the amplification. The sequence reads were validated by analysing the electropherogram data using ChromasLITE software, version 2.01 (http://technelysium.com.au/). To identify the closest known relatives, the sequences were queried with NCBI and CBS yeast nucleotide databases. The sequences obtained from both sequencing and nucleotide databases were aligned using Clustal X algorithm and a neighbour joining tree was constructed by Kimura’s evolutionary distance matrix obtained from the multiple sequence alignment using MEGA4 phylogenetic software. Bootstrap values for 1000 replicates were shown at the node of cluster branch. The sequences were deposited to NCBI GenBank under the following accession numbers: JF439366 − JF439369 and KF268351 − KF268354.

## Results

### *In silico* selection of differentiating restriction enzymes

**Figure 1:**
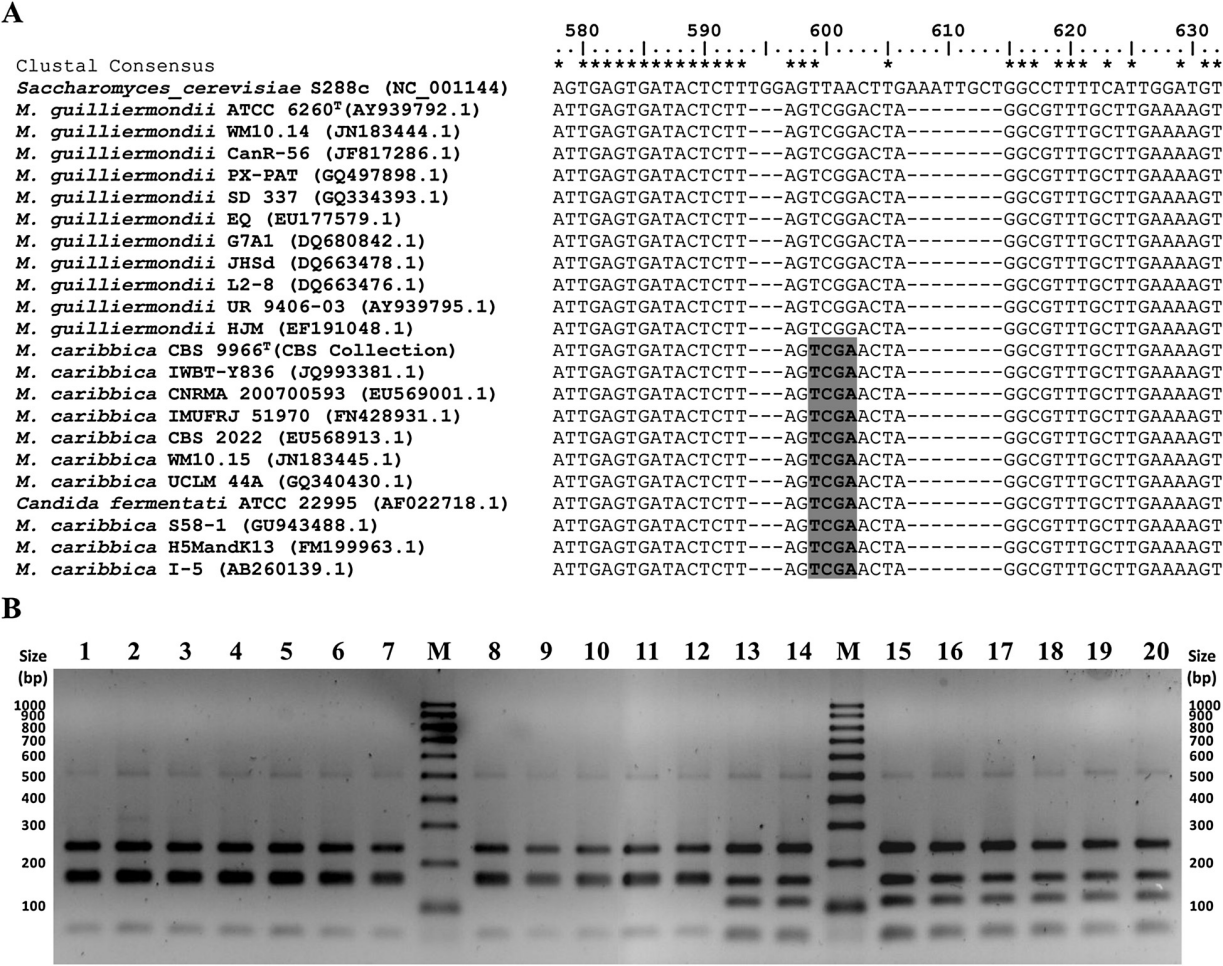
**Differentiation of *M. guilliermondii *and *M. caribbica *by *Taq*I digestion of ITS1-5.8S-ITS2. A**: Multiple sequence alignment of representative ITS1-5.8S-ITS2 sequences of the two species obtained from NCBI GenBank and CBS yeast database showing position of *Taq*I recognition site (highlighted) which distinctly differentiated the two species. **B**: *Taq*I restriction digestion profile of ITS1-5.8S-ITS2 amplicons obtained from some of the representative isolates. Lane 1: *C. guilliermondii* ATCC 6260; Lane 2 − 12: isolates of *M. guilliermondii* genotype group MG (A1S10Y1, A2S10Y1, A3S9Y1, A2S9Y1, A3S11Y1, A3S2Y1, A3S6Y1, A2S6Y1, A1S9Y1, Kw3S3Y1 and Kw2S11Y2); Lane 13 – 20: isolates of *M. caribbica* genotype group MC (A1S10Y2a, A1S10Y3, A1S10Y5, Kw3S2Y1, Kw2S3Y1, Kw3S3Y3, Kw3S3Y4 and Kw1S7Y2); Lane M: PCR 100 bp Low DNA ladder (Sigma-Aldrich).

The full length ITS1-5.8S-ITS2 sequences of *M. guilliermondii* and *M. caribbica* were retrieved from NCBI and CBS yeast nucleotide databases and subjected to multiple sequence alignment followed by *in silico* restriction digestion. Three variable regions differentiating *M. guilliermondii* from *M. caribbica* were identified. Seven restriction enzymes (*Ars*I, *Bfa*I, *Bsr*I, *Hpy*188I, *Hpy*CH4III, *Mme*I and *Taq*I) which cut the variable regions differently were identified (Figure 1A and Additional file 2: Figure S2). Considering the length and the number of polymorphic fragments with sizes greater than 100 bp (for easy analysis in normal agarose gel), *Bfa*I, *Mme*I and *Taq*I were found appropriate. Notably, commonly available *Taq*I gave distinct species-specific differentiation between the two species (Additional file 1: Table S4 and Additional file 2: Figure S3). We also tested the selected three restriction enzymes (*Bfa*I, *Mme*I and *Taq*I) for differentiating *M. guilliermondii* and *M. caribbica* from other closely related members of *M. guilliermondii* complex (Additional file 1: Table S4). Except *C. carpophila and M. caribbica,* all other members of *M. guilliermondii* complex were distinctly differentiated during the analysis.

### Evaluation of *in silico* selected restriction enzymes by *in vitro* ITS-RFLP

To validate the above *in silico* selection, the 55 yeast isolates of *M. guilliermondii* complex (which were not differentiated by phenotypic characterization and D1/D2 sequencing) were analysed by ITS-RFLP using the selected *Taq*I restriction enzyme in comparison with the type strain *C. guilliermondii* ATCC 6260. All the tested isolates and the type strain gave a single PCR amplicon of molecular size of 607 bp. As predicted by the *in silico* analysis, *Taq*I ITS-RFLP distinctly differentiated the isolates into two genotype groups. Forty seven isolates produced *M. guilliermondii*-specific pattern (MG), while the remaining eight isolates generated *M. caribbica*-specific pattern (MC) (Table 1). Examples of *Taq*I ITS-RFLP profiles differentiating the above two species are shown in Figure 1B.

**Table 1:** **Differentiation of ambiguous 55 yeast isolates obtained from *soibum *** **into *Meyerozyma guilliermondii *** **and *Meyerozyma caribbica***

**Group (Number of isolates)**	**Representative strains**	**Taxonomic designation**					
		API 20 C AUX^*^	***Taq*I-ITS-RFLP**	**Sequencing**		**mtDNA-RFLP**	**Karyotyping**
				**LSU D1/D2**	**ITS1-5.8S-ITS2**		
MG (47)	A1S10Y1, Kw2S11Y2	*M. guilliermondii*	*M. guilliermondii*	*M. guilliermondii/M. caribbica* (JF439368, JF439369)^†^	*M. guilliermondii* (KF268351, KF268352)	*M. guilliermondii*	*M. guilliermondii*
MC (08)	Kw1S7Y2, Kw3S2Y1	*M. guilliermondii*	*M. caribbica*	*M. guilliermondii/M. caribbica* (JF439366, JF439367)	*M. caribbica* (KF268353, KF268354)	*M. caribbica*	*M. caribbica*
Type strain	ATCC 6260	*M. guilliermondii*	*M. guilliermondii*	*M. guilliermondii/M. caribbica* (AJ508562.1)	*M. guilliermondii* (AY939792.1)	*M. guilliermondii*	*M. guilliermondii*

^*^No identification data for *M. caribbica* is included in the database.

^†^GenBank accession numbers.

### Validation of ITS-RFLP assay

**Figure 2:**
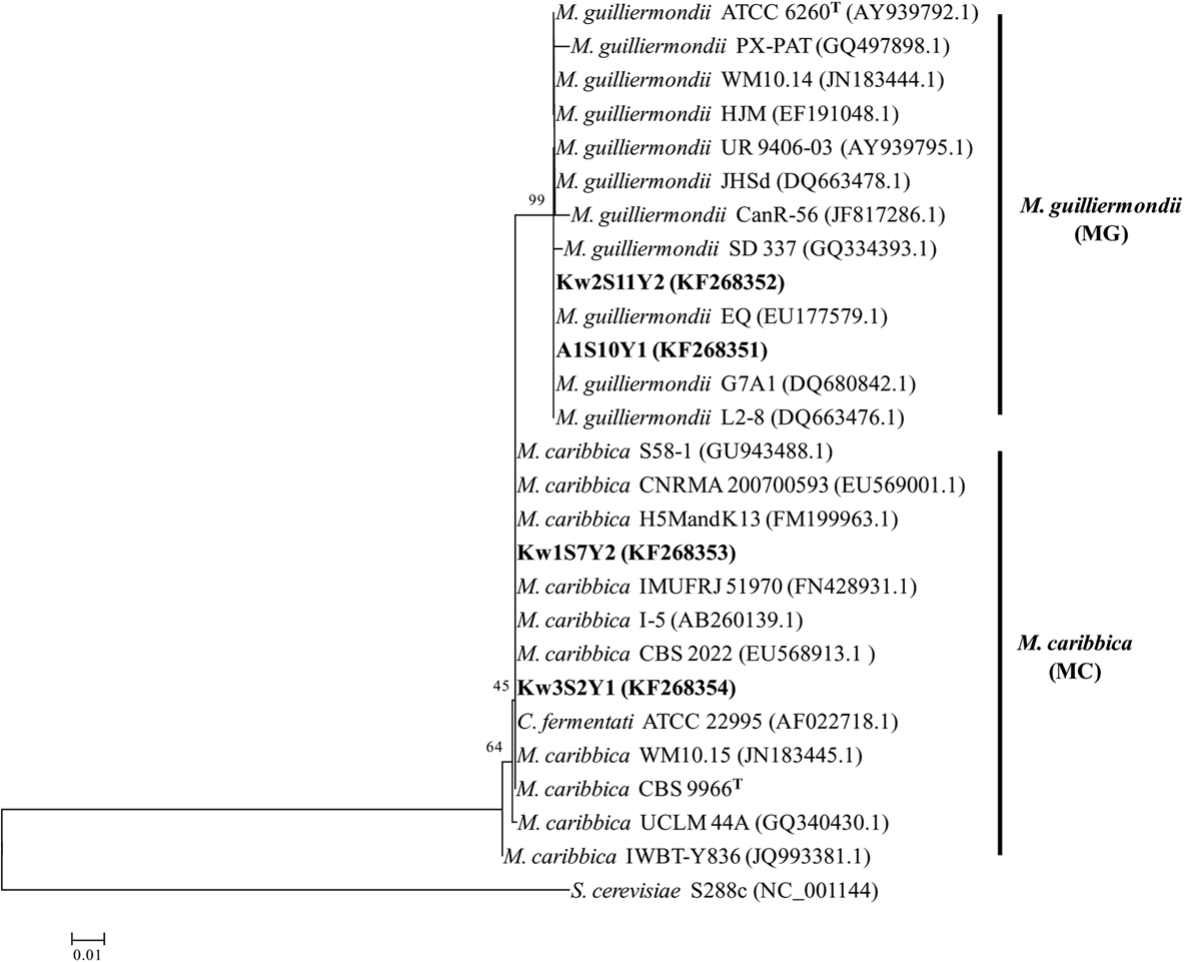
**Neighbour-joining (NJ) phylogenetic tree showing taxa-specific separation of *M. guilliermondii *from *M. caribbica.*** The tree was constructed based on the evolutionary distance calculated using Kimura-2 parameter from the nucleotide sequence of ITS1-5.8S-ITS2. The percentage of replicate trees in which the associated taxa clustered together in the bootstrap test (1000 replicates) is shown next to the branches for values >40%. The bar represents 1% sequence divergence. GenBank accession numbers are mentioned within the parentheses. *S. cerevisiae* was the outgroup in the analysis. ^T^ = Type strain.

The above ITS-RFLP based discrimination of *M. guilliermondii* and *M. caribbica* was further confirmed by ITS1-5.8S-ITS2 sequencing, mtDNA-RFLP fingerprinting and PFGE karyotyping (Table 1). The ITS sequences of the isolates in each genotype group MG and MC matched with the sequences of the type strains *C. guilliermondii* ATCC 6260 and *M. caribbica* CBS 9966 with 99.6% and 99.8% similarity respectively. The sequences between the two groups were 99% identical showing only 5 nucleotide differences which were the same as shown by the above type strain sequences. Unlike D1/D2 region, the ITS sequences formed distinct cluster of *M. guilliermondii* and *M. caribbica* during phylogenetic analysis (Figure 2). The ITS sequences of *M. guilliermondii* strains PX-PAT, CanR-56 and SD 337; *M. caribbica* strains UCLM 44A and IWBT-Y836 showed 1 – 3 nucleotide differences which was reflected as divergence within the clusters. The mean evolutionary divergence of 0.0131 between the two clusters was 6 times more than the divergence within each cluster.

**Figure 3:**
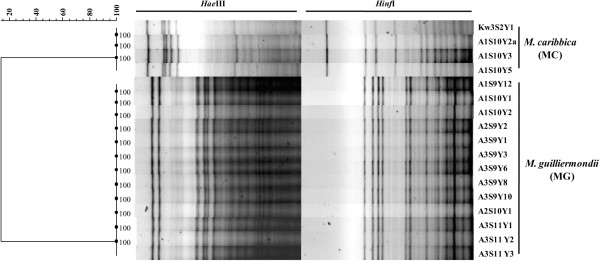
**mtDNA-RFLP based dendrogram showing distinct clustering of *M. guilliermondii *and *M. caribbica*.** The dendrogram was constructed using UPGMA algorithm on Jaccard similarity coefficients generated from *Hae*III and *Hinf*I restriction digestion profile of mtDNA of some of the representative isolates. Value at each branch node indicates the branch quality with 1000 bootstrap replications. The scale represents the similarity.

**Figure 4:**
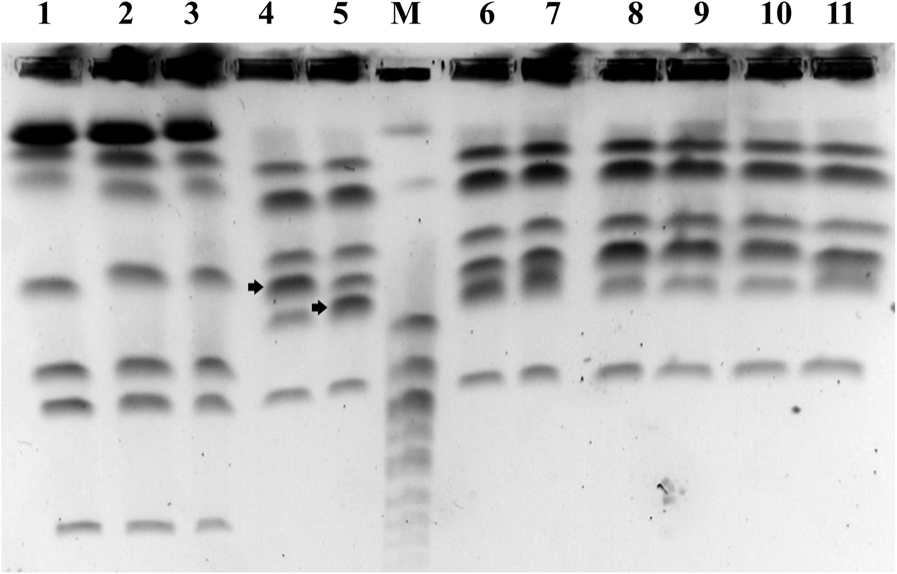
**PFGE karyotype patterns of isolates belonging to *M. guilliermondii *and *M. caribbica *genotype groups.** Lane 1: *C. guilliermondii* ATCC 6260; Lane 2 − 3: *M. guilliermondii* isolates A1S10Y1 and Kw2S11Y2; Lane 4 − 11: *M. caribbica* isolates A1S10Y2a, A1S10Y3, A1S10Y5, Kw3S2Y1, Kw2S3Y1, Kw3S3Y3, Kw3S3Y4 and Kw1S7Y2; Lane M: *S. cerevisiae* PFGE marker (Sigma-Aldrich). Right arrow indicates the co-migrating chromosomal doublets showing strain level diversity.

The mtDNA-RFLP using *Hae*III and *Hinf*I distinctly segregated the yeast isolates into *M. guilliermondii* and *M. caribbica*. mtDNA-RFLP profile-based dendrogram formed two clusters (Figure 3) similar to the ITS-RFLP groups. Between the two enzymes used, *Hinf*I showed higher polymorphism than *Hae*III. Electrophoretic karyotyping also distinctly discriminated the above two species (Figure 4). The species-specific mtDNA-RFLP pattern suggested that the isolates of each group belonged to only one strain (Figure 3). Whereas electrophoretic karyotyping brought out strain level diversity in both the groups which confirmed that multiple strains of *M. guilliermondii* and *M. caribbica* were involved in the indigenous bamboo shoot fermentation (Figure 4 and Additional file 2: Figure S4).

## Discussion

In recent times, the frequency of emerging infectious diseases caused by the opportunistic yeast species of NAC and non-*Candida* groups has increased in immunosuppressed patients [12,44]. This is linked with the indiscriminate use of broad-spectrum antifungal drugs and global climate change [45-47]. Most of these closely related yeast species are often misidentified by the conventional phenotypic, biochemical and antibiotic susceptibility methods. Thus, accurate identification of these species has become essential for clinical management and effective antifungal therapy.

In the present study, we showed that the developed ITS-RFLP method was reliable and consistent for distinct differentiation of closely related *M. guilliermondii* from *M. caribbica* for which phenotypic methods and D1/D2 sequencing were inconclusive. Our results also indicated that sequencing of both D1/D2 and ITS regions will increase the resolution of species identification which can be further improved by multigene sequence-based phylogenetic approach [3,48,49]. However, the presence of incorrectly identified, insufficiently annotated and non-updated entries in the public nucleotide databases may underestimate the resolving power of these taxonomic markers [50]. Out of the 29 sequences of LSU rRNA gene for *M. guilliermondii* available in NCBI GenBank, 17 sequences (58.62%) clustered with *M. caribbica* type strain CBS 9966 [GenBank: EU348786] (Additional file 2: Figure S1).

The choice of appropriate restriction endonucleases is critical for RFLP experiments. The commonly used *Cfo*I, *Hae*III and *Hinf*I [37,41] failed to segregate *M. guilliermondii* from other species of the same genus during *in silico* and *in vitro* ITS-RFLP analysis. Our results indicated that *in silico* selection of restriction enzymes using the publicly available sequences from various strains of the target species is a better approach than randomly selecting the previously described and commonly used enzymes. This approach has been proven to be highly effective and reproducible [36,51-53], and many online resources have been developed for this purpose [54-57]. Clinical isolates of *Candida famata* and *Candida palmioleophila* were also frequently misidentified as *M. guilliermondii*[30,31]. *In silico* analysis confirmed that the developed ITS-RFLP method can also discriminate these species (data not shown). This *in silico* selection approach can be effectively applied to other cryptic yeast species of clinical importance for the development of RFLP based diagnostic tools.

The developed method of ITS-RFLP using *Taq*I differentiated *M. guilliermondii* and *M. caribbica* at species level. This method is simple, rapid and reliable in comparison to the commonly used sequencing methods. The entire analysis starting from DNA extraction to ITS-RFLP profiling can be completed within 8 h. Further studies using higher number of strains of these two species from different clinical sources are required to confirm the robustness of this method for diagnostic applications. Though the combination of ITS-RFLP profiles generated by *Taq*I, *Bfa*I and *Mme*I differentiated other closely related species of the *M. guilliermondii* complex from *M. guilliermondii* and *M. caribbica* during *in silico* analysis, it is yet to be confirmed through *in vitro* analysis using reference strains.

In our present study the use of either *Hae*III or *Hinf*I for mtDNA-RFLP was sufficient to differentiate the two species, but failed to give strain level differentiation even when both enzymes were used. This demonstrated that mtDNA-RFLP can also be used for distinct differentiation of closely related species. The *Hinf*I mtDNA-RFLP pattern of our *M. guilliermondii* isolates was similar with the mtDNA restriction pattern ‘E’ of *M. guilliermondii* strains isolated from wineries in Alentejo, Portugal [58]. This genotype was linked with the production of flavour compound, 4-ethylphenol in wine. The major phenolic flavour compound (4-methylphenol) detected from fermented bamboo shoot product, *soibum* (Singh NR: unpublished observations) might also have originated from *M. guilliermondii*. Future study is required to characterize the flavour compound producing strain for starter culture development. Though fresh bamboo shoots are highly perishable, the fermented bamboo shoot can be preserved up to one year after fermentation without any deterioration or change in its organoleptic character. This long term preservation may be linked with the dominant presence of *M. guilliermondii* which has been reported as an efficient biological control agent [24,25]. Being an emerging infectious yeast, the presence of *M. guilliermondii* in fermented food is a great concern regarding the safety of its consumption. Further study in strain level is required to unravel the pathogenic potential of *M. guilliermondii* associated with *soibum* fermentation.

## Conclusions

In this study, we described an ITS-RFLP method developed through an integrated approach of *in silico* selection of restriction enzymes and *in vitro* validations for distinct differentiation of frequently misidentified *M. guilliermondii* from *M. caribbica*, which can be used as an alternative or an adjunct to ITS sequencing. This method may be used for rapid and accurate identification of emerging infectious yeasts of the *Saccharomycotina* CTG clade. This approach can also be used for other closely related species complex when phenotypic methods and D1/D2 sequencing are ambiguous.

## Competing interests

The authors declare that they have no competing interests.

## Authors’ contributions

WR and KJ conceived and designed the study, carried out the analysis and interpretation of the data and drafted the manuscript. WR carried out the molecular studies, performed the phenotypic identification and executed the *in silico* and sequence analyses. SK contributed to the molecular studies. GA and KJ critically revised the draft manuscript. All authors read and approved the final manuscript.

## Supplementary Material

Additional file 1: Table S1 List of the 55 yeast isolates used in the present study. **Table S2.** Carbon substrate assimilation pattern of representative strains of *M. guilliermondii* complex using API 20 C AUX yeast identification system. **Table S3.** Taxonomic assignment of isolates belonging to *M. guilliermondii* complex by sequencing of LSU rRNA gene D1/D2 domain. **Table S4.** List of the selected type-II restriction endonucleases that differentiated *M. guilliermondii* from *M. caribbica* and other species of *M. guilliermondii* complex during *in silico* restriction digestion of the ITS1-5.8S-ITS2 amplicon sequences. Click here for file

Additional file 2: Figure S1 Neighbour-joining phylogenetic tree based on LSU rRNA gene D1/D2 sequences showing taxa-nonspecific segregation of *M. guilliermondii* strains. The tree was constructed based on the evolutionary distance calculated using Kimura-2 parameter from the representative nucleotide sequences of *M. guilliermondii* and *M. caribbica* (position 13 to 308 of LSU rRNA gene of *S. cerevisiae* CBS 1171, GenBank Accession No. AY048154.1). The percentage of replicate trees in which the associated taxa clustered together in the bootstrap test (1000 replicates) is shown next to the branches. The bar represents 1% sequence divergence. GenBank accession numbers are mentioned within the parentheses. *S. cerevisiae* was the outgroup in the analysis. ^T^ = Type strain. **Figure S2. ***In silico* identified restriction enzymes which distinctly differentiated *M. guilliermondii* from *M. caribbica*. Multiple sequence alignment of representative ITS1-5.8S-ITS2 sequences of various strains of the two species obtained from NCBI GenBank and CBS yeast database showing position of identified *Ars*I (A), *Bfa*I (B), *Bsr*I (C), *Hpy*188I (D), *Hpy*CH4III (E), and *Mme*I (F) restriction recognition sites (highlighted) which distinctly differentiated the two species. The nucleotide position was based on the sequence of the *in silico* PCR amplicon of ITS1-5.8S-ITS2 of *S. cerevisiae* strain S288c (NC_001144) including gaps generated during multiple sequence alignment. *C. fermentati* is the anamorph of *M. caribbica*. ^T^ = Type strain. **Figure S3. ***In silico* restriction digestion profile of *M. guilliermondii* and *M. caribbica* ITS1-5.8S-ITS2 amplicon. The theoretical restriction digestion profile was generated using NEBcutter, version 2.0 (http://tools.neb.com/NEBcutter2/). Lane G: *M. guilliermondii* ATCC 6260; Lane C: *M. caribbica* CBS 9966; Lane M: 100 bp DNA ladder. **Figure S4.** Strain level diversity of *M. guilliermondii* revealed by PFGE karyotyping. Lane 1 − 13: Isolates A3S2Y1, Kw1S2Y1, Kw3S3Y1, A3S6Y1, A2S6Y1, A1S9Y1, A1S9Y5, A2S9Y1, A3S9Y1, A3S9Y9, A2S10Y1, A2S10Y4 and A3S11Y1. White arrow indicates the polymorphic chromosomal band. Click here for file
