# Supplementary material for: Reliable differentiation of Meyerozyma guilliermondii from Meyerozyma caribbica by internal transcribed spacer restriction fingerprinting
Source: BMC Microbiol. 2014 Feb 28;14:52. doi: 10.1186/1471-2180-14-52 (PMC3946169; doi:10.1186/1471-2180-14-52)
Supplement: Additional file 1: Table S1 — List of the 55 yeast isolates used in the present study. Table S2. Carbon substrate assimilation pattern of representative strains of M. guilliermondii complex using API 20 C AUX yeast identification system. Table S3. Taxonomic assignment of isolates belonging to M. guilliermondii complex by sequencing of LSU rRNA gene D1/D2 domain. Table S4. List of the selected type-II restriction endonucleases that differentiated M. guilliermondii from M. caribbica and other species of M. guilliermondii complex during in silico restriction digestion of the ITS1-5.8S-ITS2 amplicon sequences. [file 1471-2180-14-52-S1.pdf]

Supplemental Tables

Table S1 List of the 55 yeast isolates used in the present study

Place of Collection	Production Lot	Stage of collection (days of indigenous fermentation)	Isolates
Andro (N24°44′ E94°02′, 810 msl), Imphal-East, Manipur, India	A1	63	A1S9Y1, A1S9Y2, A1S9Y3, A1S9Y4, A1S9Y5, A1S9Y6, A1S9Y7, A1S9Y8, A1S9Y9, A1S9Y10, A1S9Y11, A1S9Y12
		108	A1S10Y1, A1S10Y2, A1S10Y2a, A1S10Y3, A1S10Y4, A1S10Y5
	A2	28	A2S6Y1
		63	A2S9Y1, A2S9Y2, A2S9Y3
		108	A2S10Y1, A2S10Y3, A2S10Y4, A2S10Y5, A2S10Y6
	A3	2	A3S2Y1
		28	A3S6Y1, A3S6Y2
		63	A3S9Y1, A3S9Y2, A3S9Y3, A3S9Y4, A3S9Y5, A3S9Y6, A3S9Y7, A3S9Y8, A3S9Y9, A3S9Y10
		153	A3S11Y1, A3S11 Y2, A3S11 Y3
Kwatha (N24°19′	Kw1	2	Kw1S2Y1
		35	Kw1S7Y2
	Kw2	7	Kw2S3Y1

E94°16′, 474 msl), Chandel, Manipur, India		153	Kw2S11Y1, Kw2S11Y2, Kw2S11Y3
	Kw3	2	Kw3S2Y1
		7	Kw3S3Y1, Kw3S3Y2, Kw3S3Y3, Kw3S3Y4, Kw3S3Y5

Table S2 Carbon substrate assimilation pattern of representative strains of *M. guilliermondii* complex using API 20 C AUX yeast identification system

Sl. No.	Strain No.	API 20 C AUX carbon substrates[1]																			Identification
		D-Glucose	Glycerol	Calcium 2-keto-gluconate	L-Arabinose	D-Xylose	Adonitol	Xylitol	D-Galactose	Inositol	D-Sorbitol	Methyl-αD-glucopyranoside	N-Acetyl-glucosamine	D-Cellobiose	D-Lactose	D-Maltose	D-Saccharose	D-Trehalose	D-Melezitose	D-Raffinose	
1.	A1S10Y1	+	+	+	+	+	+	+	+	-	+	+	+	+	-	+	+	+	+	+	*M. guilliermondii*
2.	Kw2S11Y2	+	+	+	+	+	+	+	+	-	+	+	+	+	-	+	+	+	+	+	*M. guilliermondii*
3.	Kw1S7Y2	+	+	+	+	+	+	+	+	-	+	+	+	+	-	+	+	+	+	+	*M. guilliermondii*
4.	Kw3S2Y1	+	+	+	+	+	+	+	+	-	+	+	+	-	-	+	+	+	+	+	*M. guilliermondii*
5.	A1S10Y3	+	+	+	+	+	+	+	+	-	+	+	+	+	-	+	+	+	+	+	*M. guilliermondii*
6.	Kw2S3Y1	+	+	+	+	+	+	+	+	-	+	+	+	+	-	+	+	+	+	+	*M. guilliermondii*
7.	ATCC	+	+	+	+	+	+	+	+	-	+	+	+	+	-	+	+	+	+	+	*M. guilliermondii*

	6260[2]																				

[1]Reading was made after 72 hours of inoculation. Growth in the cupule, i.e., more turbid than the negative control (no carbon substrate) is indicated by '+' sign and no growth in the cupule by '-'sign.

[2]Type strain

Table S3 Taxonomic assignment of isolates belonging to *M. guilliermondii* complex by sequencing of LSU rRNA gene D1/D2 domain

Representative Strains	Size (bp)	Score	Sequence coverage (%)	Max. Identity %	No. of matched sequences with the same score, coverage and identity (% of matched sequences)		GenBank Accession Number
					M. guilliermondii	*M. caribbica*	
A1S10Y1	358	656	100	99 (358/359)	67 (70.5)	28 (29.5)	JF439368
Kw2S11Y2	306	566	100	100 (306/306)	03 (27.3)	08 (72.7)	JF439369
Kw1S7Y2	394	728	100	100 (394/394)	67 (69.8)	29 (30.2)	JF439366
Kw3S2Y1	577	1053	99	99 (572/573)	01 (12.5)	07 (87.5)	JF439367

Table S4 List of the selected type-II restriction endonucleases that differentiated *M. guilliermondii* from *M. caribbica* and other species of *M. guilliermondii* complex during *in silico* restriction digestion of the ITS1-5.8S-ITS2 amplicon sequences

**Sl. No.**	**Restriction enzyme**	**Recognition sequence**	**No. of cutting sites***							**Cut positions**							**Theoretical restriction fragment size (bp)**						
			Mg	Mc	Cc	Cs	Ca	Ce	Cg	Mg	Mc	Cc	Cs	Ca	Ce	Cg	Mg	Mc	Cc	Cs	Ca	Ce	Cg
1.	*Ars*I	▲$(N)_5$▼ $(N)_8$GAC$(N)_6$TTYG$(N)_6$▲$(N)_5$▼	1	0	0	0	1	0	NA	393/388, 425/420	-	-	-	395/390, 427/422	-	NA	393, 182, 32	-	-	-	395, 182, 32	-	NA
2.	*Bfa*I	C▼TA▲G	3	2	2	3	3	2	NA	118/120, 404/406, 441/443	118/120, 404/406	117/119, 403/405	118/120, 404/406, 441/443	118/120, 406/408, 443/445	119/121, 446/448	NA	286, 166, 118, 37	286, 203, 118	286, 203, 117	286, 166, 118, 37	288, 166, 118, 37	327, 167, 119	NA
3.	*Bsr*I	ACTG▲GN▼	0	1	1	0	0	0	NA	-	445/443	444/442	-	-	-	NA	-	445, 162	444, 162	-	-	-	NA
4.	*Hpy*188I	TC▲N▼GA	1	0	0	0	1	0	NA	401/400	-	-	-	403/402	-	NA	401, 206	-	-	-	403, 206	-	NA

5.	*Hpy*CH4III	AC▴N▾GT	3	2	2	2	3	2	NA	32/31, 80/79, 171/170	32/31, 80/79	32/31, 79/78	32/31, 80/79	32/31, 80/79, 176/175	32/31, 80/79	NA	436, 91, 48, 32	527, 48, 32	527, 47, 32	527, 48, 32	433, 96, 48, 32	533, 48, 32	NA
6.	*Mme*I	TCCRAC(N)$_{18}$ ▴NN▾	2	1	1	1	1	1	NA	379/377, 504/502	504/502	503/501	504/502	506/504	510/508	NA	379, 125, 103	504, 103	503, 103	504, 103	506, 103	510, 103	NA
7.	*Taq*I	T▾CG▴A	3	4	4	4	3	2	NA	232/234, 291/293, 454/456	232/234, 291/293, 399/401, 454/456	231/233, 290/292, 398/400, 453/455	232/234, 291/293, 399/401, 454/456	234/236, 293/295, 456/458	237/239, 296/298,	NA	232, 163, 153, 59	232, 153, 108, 59, 55	231, 153, 108, 59, 55	232, 153, 108, 59, 55	234, 163, 153, 59	317, 237, 59	NA

*Mg: *Meyerozyma guilliermondii* ATCC 6260 (Type) (GenBank accession number: AY939792.1), Mc: *Meyerozyma caribbica* CBS 9966 (Type) (sequence was obtained from CBS collection), Cc: *Candida carpophila* CBS 5256 (Type) (sequence was obtained from CBS collection), Cs: *Candida smithsonii* ATCC MYA-4323 (Type) (GenBank accession number: FJ172249.1), Ca: *Candida athensensis* ATCC MYA-4324

(Type) (GenBank accession number: FJ172250.1), Ce: *Candida elateridarum* ATCC MYA-4325 (Type) (GenBank accession number: FJ196772.1), Cg: *Candida glucosophila*, NA: no ITS1-5.8S-ITS2 sequence is available till date.
