# Supplementary material for: Reliable differentiation of Meyerozyma guilliermondii from Meyerozyma caribbica by internal transcribed spacer restriction fingerprinting
Source: BMC Microbiol. 2014 Feb 28;14:52. doi: 10.1186/1471-2180-14-52 (PMC3946169; doi:10.1186/1471-2180-14-52)

Figure S1

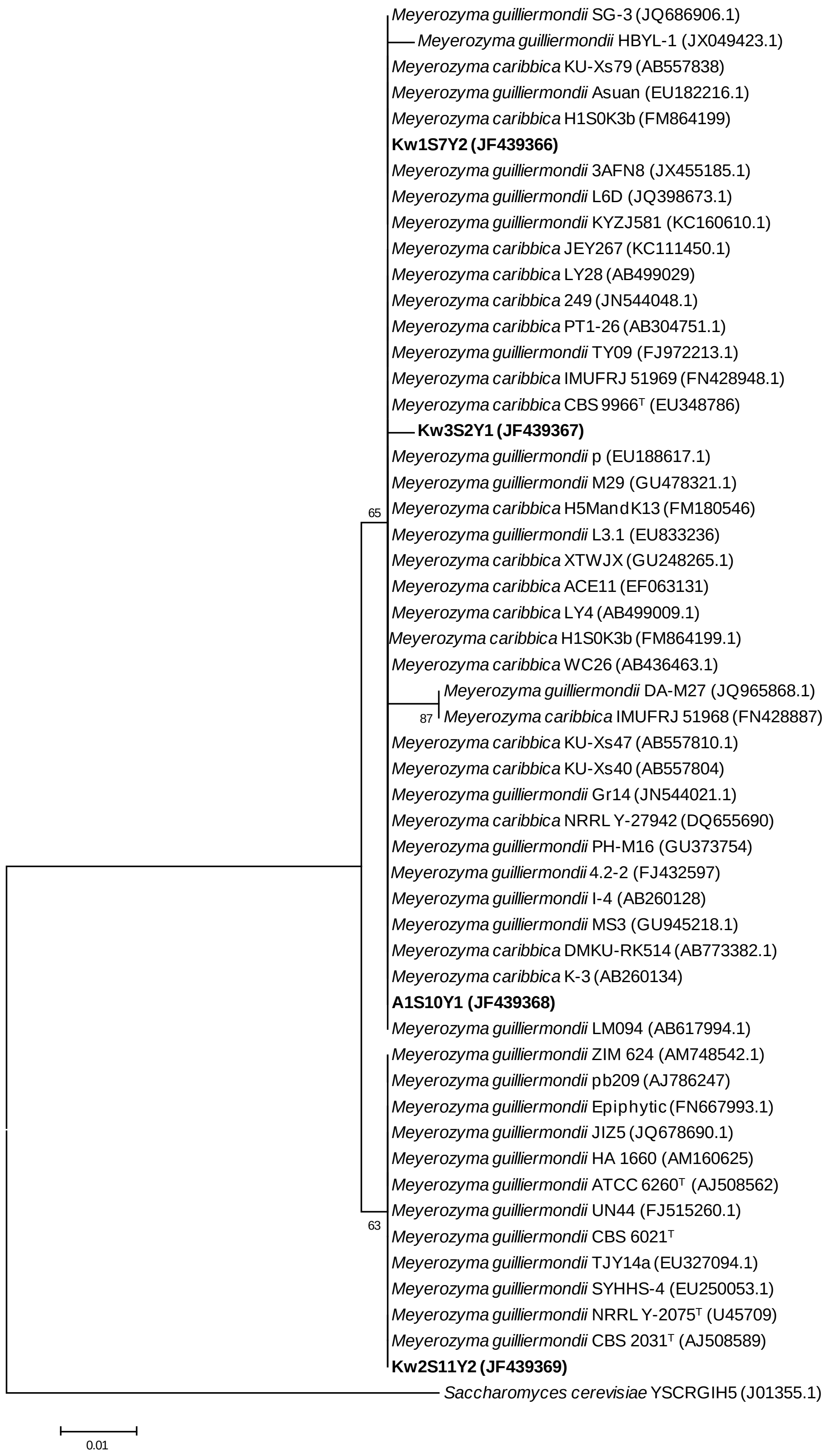

Meyerozyma guilliermondii SG-3 (JQ686906.1)
Meyerozyma guilliermondii HBYL-1 (JX049423.1)
Meyerozyma caribbica KU-Xs79 (AB557838)
Meyerozyma guilliermondii Asuan (EU182216.1)
Meyerozyma caribbica H1S0K3b (FM864199)
Kw1S7Y2 (JF439366)
Meyerozyma guilliermondii 3AFN8 (JX455185.1)
Meyerozyma guilliermondii L6D (JQ398673.1)
Meyerozyma guilliermondii KYZJ581 (KC160610.1)
Meyerozyma caribbica JEY267 (KC111450.1)
Meyerozyma caribbica LY28 (AB499029)
Meyerozyma caribbica 249 (JN544048.1)
Meyerozyma caribbica PT1-26 (AB304751.1)
Meyerozyma guilliermondii TY09 (FJ972213.1)
Meyerozyma caribbica IMUFRJ 51969 (FN428948.1)
Meyerozyma caribbica CBS 9966^{T} (EU348786)
Kw3S2Y1 (JF439367)
Meyerozyma guilliermondii p (EU188617.1)
Meyerozyma guilliermondii M29 (GU478321.1)
65
Meyerozyma caribbica H5MandK13 (FM180546)
Meyerozyma guilliermondii L3.1 (EU833236)
Meyerozyma caribbica XTWJX (GU248265.1)
Meyerozyma caribbica ACE11 (EF063131)
Meyerozyma caribbica LY4 (AB499009.1)
Meyerozyma caribbica H1S0K3b (FM864199.1)
Meyerozyma caribbica WC26 (AB436463.1)
Meyerozyma guilliermondii DA-M27 (JQ965868.1)
87
Meyerozyma caribbica IMUFRJ 51968 (FN428887)
Meyerozyma caribbica KU-Xs47 (AB557810.1)
Meyerozyma caribbica KU-Xs40 (AB557804)
Meyerozyma guilliermondii Gr14 (JN544021.1)
Meyerozyma caribbica NRRL Y-27942 (DQ655690)
Meyerozyma guilliermondii PH-M16 (GU373754)
Meyerozyma guilliermondii 4.2-2 (FJ432597)
Meyerozyma guilliermondii I-4 (AB260128)
Meyerozyma guilliermondii MS3 (GU945218.1)
Meyerozyma caribbica DMKU-RK514 (AB773382.1)
Meyerozyma caribbica K-3 (AB260134)
A1S10Y1 (JF439368)
Meyerozyma guilliermondii LM094 (AB617994.1)
Meyerozyma guilliermondii ZIM 624 (AM748542.1)
Meyerozyma guilliermondii pb209 (AJ786247)
Meyerozyma guilliermondii Epiphytic (FN667993.1)
Meyerozyma guilliermondii JIZ5 (JQ678690.1)
Meyerozyma guilliermondii HA 1660 (AM160625)
Meyerozyma guilliermondii ATCC 6260^{T} (AJ508562)
Meyerozyma guilliermondii UN44 (FJ515260.1)
63
Meyerozyma guilliermondii CBS 6021^{T}
Meyerozyma guilliermondii TJY14a (EU327094.1)
Meyerozyma guilliermondii SYHHS-4 (EU250053.1)
Meyerozyma guilliermondii NRRL Y-2075^{T} (U45709)
Meyerozyma guilliermondii CBS 2031^{T} (AJ508589)
Kw2S11Y2 (JF439369)
Saccharomyces cerevisiae YSCRGIH5 (J01355.1)
0.01

Figure S2

A

```
                                                           590       600       610       620       630
                                                        ...|....|....|....|....|....|....|....|....|....|....|...
Clustal Consensus                                       ************   ***     *         *** *** * *   * ** **
Saccharomyces_cerevisiae S288c (NC_001144)              AGTGATACTCTTTGGAGTTAACTTGAAATTGCTGGCCTTTTCATTGGATGTTTTTTT
M. guilliermondii ATCC 6260T(AY939792.1)                AGTGATACTCTT---AGTCGGACTA--------GGCGTTTGCTTGAAAAGTATT---
M. guilliermondii WM10.14 (JN183444.1)                  AGTGATACTCTT---AGTCGGACTA--------GGCGTTTGCTTGAAAAGTATT---
M. guilliermondii CanR-56 (JF817286.1)                  AGTGATACTCTT---AGTCGGACTA--------GGCGTTTGCTTGAAAAGTATT---
M. guilliermondii PX-PAT (GQ497898.1)                   AGTGATACTCTT---AGTCGGACTA--------GGCGTTTGCTTGAAAAGTATT---
M. guilliermondii SD 337 (GQ334393.1)                   AGTGATACTCTT---AGTCGGACTA--------GGCGTTTGCTTGAAAAGTATT---
M. guilliermondii EQ (EU177579.1)                       AGTGATACTCTT---AGTCGGACTA--------GGCGTTTGCTTGAAAAGTATT---
M. guilliermondii G7A1 (DQ680842.1)                     AGTGATACTCTT---AGTCGGACTA--------GGCGTTTGCTTGAAAAGTATT---
M. guilliermondii JHSd (DQ663478.1)                     AGTGATACTCTT---AGTCGGACTA--------GGCGTTTGCTTGAAAAGTATT---
M. guilliermondii L2-8 (DQ663476.1)                     AGTGATACTCTT---AGTCGGACTA--------GGCGTTTGCTTGAAAAGTATT---
M. guilliermondii UR 9406-03 (AY939795.1)               AGTGATACTCTT---AGTCGGACTA--------GGCGTTTGCTTGAAAAGTATT---
M. guilliermondii HJM (EF191048.1)                      AGTGATACTCTT---AGTCGGACTA--------GGCGTTTGCTTGAAAAGTATT---
M. caribbica CBS 9966T(CBS Collection)                  AGTGATACTCTT---AGTCGAACTA--------GGCGTTTGCTTGAAAAGTATT---
M. caribbica IWBT-Y836 (JQ993381.1)                     AGTGATACTCTT---AGTCGAACTA--------GGCGTTTGCTTGAAAAGTATT---
M. caribbica CNRMA 200700593 (EU569001.1)               AGTGATACTCTT---AGTCGAACTA--------GGCGTTTGCTTGAAAAGTATT---
M. caribbica IMUFRJ 51970 (FN428931.1)                  AGTGATACTCTT---AGTCGAACTA--------GGCGTTTGCTTGAAAAGTATT---
M. caribbica CBS 2022 (EU568913.1)                      AGTGATACTCTT---AGTCGAACTA--------GGCGTTTGCTTGAAAAGTATT---
M. caribbica WM10.15 (JN183445.1)                       AGTGATACTCTT---AGTCGAACTA--------GGCGTTTGCTTGAAAAGTATT---
M. caribbica UCLM 44A (GQ340430.1)                      AGTGATACTCTT---AGTCGAACTA--------GGCGTTTGCTTGAAAAGTATT---
Candida fermentati ATCC 22995 (AF022718.1)             AGTGATACTCTT---AGTCGAACTA--------GGCGTTTGCTTGAAAAGTATT---
M. caribbica S58-1 (GU943488.1)                         AGTGATACTCTT---AGTCGAACTA--------GGCGTTTGCTTGAAAAGTATT---
M. caribbica H5MandK13 (FM199963.1)                     AGTGATACTCTT---AGTCGAACTA--------GGCGTTTGCTTGAAAAGTATT---
M. caribbica I-5 (AB260139.1)                          AGTGATACTCTT---AGTCGAACTA--------GGCGTTTGCTTGAAAAGTATT---
```

B

```
                                                        631     640       650       660       670       680  685
                                                        ....|....|....|....|....|....|....|....|....|....|....|
Clustal Consensus                                       ** **          *  ***    **   *       **    *  *  *
Saccharomyces_cerevisiae S288c (NC_001144)              GTTTTTTTTCCAAAGAGAGGTTTCTCTGCGTGCTTGAGGTATAATGCAAGTACGG
M. guilliermondii ATCC 6260T(AY939792.1)                GTATT-------GGCATGGGTAGTACTAGATA------GTGCTGTCGACCTCTCA
M. guilliermondii WM10.14 (JN183444.1)                  GTATT-------GGCATGGGTAGTACTAGATA------GTGCTGTCGACCTCTCA
M. guilliermondii CanR-56 (JF817286.1)                  GTATT-------GGCATGGGTAGTACTAGATA------GTGCTGTCGACCTCTCA
M. guilliermondii PX-PAT (GQ497898.1)                   GTATT-------GGCATGGGTAGTACTAGATA------GTGCTGTCGACCTCTCA
M. guilliermondii SD 337 (GQ334393.1)                   GTATT-------GGCATGGGTAGTACTAGATA------GTGCTGTCGACCTCTCA
M. guilliermondii EQ (EU177579.1)                       GTATT-------GGCATGGGTAGTACTAGATA------GTGCTGTCGACCTCTCA
M. guilliermondii G7A1 (DQ680842.1)                     GTATT-------GGCATGGGTAGTACTAGATA------GTGCTGTCGACCTCTCA
M. guilliermondii JHSd (DQ663478.1)                     GTATT-------GGCATGGGTAGTACTAGATA------GTGCTGTCGACCTCTCA
M. guilliermondii L2-8 (DQ663476.1)                     GTATT-------GGCATGGGTAGTACTAGATA------GTGCTGTCGACCTCTCA
M. guilliermondii UR 9406-03 (AY939795.1)               GTATT-------GGCATGGGTAGTACTAGATA------GTGCTGTCGACCTCTCA
M. guilliermondii HJM (EF191048.1)                      GTATT-------GGCATGGGTAGTACTAGATA------GTGCTGTCGACCTCTCA
M. caribbica CBS 9966T(CBS Collection)                  GTATT-------GGCATGGGTAGTACTGGATA------GTGCTGTCGACCTCTCA
M. caribbica IWBT-Y836 (JQ993381.1)                     GTATT-------GGCATGGGTAGTACTGGATA------GTGCTGTCGACCTCTCA
M. caribbica CNRMA 200700593 (EU569001.1)               GTATT-------GGCATGGGTAGTACTGGATA------GTGCTGTCGACCTCTCA
M. caribbica IMUFRJ 51970 (FN428931.1)                  GTATT-------GGCATGGGTAGTACTGGATA------GTGCTGTCGACCTCTCA
M. caribbica CBS 2022 (EU568913.1)                      GTATT-------GGCATGGGTAGTACTGGATA------GTGCTGTCGACCTCTCA
M. caribbica WM10.15 (JN183445.1)                       GTATT-------GGCATGGGTAGTACTGGATA------GTGCTGTCGACCTCTCA
M. caribbica UCLM 44A (GQ340430.1)                      GTATT-------GGCATGGGTAGTACTGGATA------GTGCTGTCGACCTCTCA
Candida fermentati ATCC 22995 (AF022718.1)             GTATT-------GGCATGGGTAGTACTGGATA------GTGCTGTCGACCTCTCA
M. caribbica S58-1 (GU943488.1)                         GTATT-------GGCATGGGTAGTACTGGATA------GTGCTGTCGACCTCTCA
M. caribbica H5MandK13 (FM199963.1)                     GTATT-------GGCATGGGTAGTACTGGATA------GTGCTGTCGACCTCTCA
M. caribbica I-5 (AB260139.1)                          GTATT-------GGCATGGGTAGTACTGGATA------GTGCTGTCGACCTCTCA
```

C

```
                                                        631     640       650       660       670       680  685
                                                        ....|....|....|....|....|....|....|....|....|....|....|
Clustal Consensus                                       ** **          *  ***    **   *       **    *  *  *
Saccharomyces_cerevisiae S288c (NC_001144)              GTTTTTTTTCCAAAGAGAGGTTTCTCTGCGTGCTTGAGGTATAATGCAAGTACGG
M. guilliermondii ATCC 6260T(AY939792.1)                GTATT-------GGCATGGGTAGTACTAGATA------GTGCTGTCGACCTCTCA
M. guilliermondii WM10.14 (JN183444.1)                  GTATT-------GGCATGGGTAGTACTAGATA------GTGCTGTCGACCTCTCA
M. guilliermondii CanR-56 (JF817286.1)                  GTATT-------GGCATGGGTAGTACTAGATA------GTGCTGTCGACCTCTCA
M. guilliermondii PX-PAT (GQ497898.1)                   GTATT-------GGCATGGGTAGTACTAGATA------GTGCTGTCGACCTCTCA
M. guilliermondii SD 337 (GQ334393.1)                   GTATT-------GGCATGGGTAGTACTAGATA------GTGCTGTCGACCTCTCA
M. guilliermondii EQ (EU177579.1)                       GTATT-------GGCATGGGTAGTACTAGATA------GTGCTGTCGACCTCTCA
M. guilliermondii G7A1 (DQ680842.1)                     GTATT-------GGCATGGGTAGTACTAGATA------GTGCTGTCGACCTCTCA
M. guilliermondii JHSd (DQ663478.1)                     GTATT-------GGCATGGGTAGTACTAGATA------GTGCTGTCGACCTCTCA
M. guilliermondii L2-8 (DQ663476.1)                     GTATT-------GGCATGGGTAGTACTAGATA------GTGCTGTCGACCTCTCA
M. guilliermondii UR 9406-03 (AY939795.1)               GTATT-------GGCATGGGTAGTACTAGATA------GTGCTGTCGACCTCTCA
M. guilliermondii HJM (EF191048.1)                      GTATT-------GGCATGGGTAGTACTAGATA------GTGCTGTCGACCTCTCA
M. caribbica CBS 9966T(CBS Collection)                  GTATT-------GGCATGGGTAGTACTGGATA------GTGCTGTCGACCTCTCA
M. caribbica IWBT-Y836 (JQ993381.1)                     GTATT-------GGCATGGGTAGTACTGGATA------GTGCTGTCGACCTCTCA
M. caribbica CNRMA 200700593 (EU569001.1)               GTATT-------GGCATGGGTAGTACTGGATA------GTGCTGTCGACCTCTCA
M. caribbica IMUFRJ 51970 (FN428931.1)                  GTATT-------GGCATGGGTAGTACTGGATA------GTGCTGTCGACCTCTCA
M. caribbica CBS 2022 (EU568913.1)                      GTATT-------GGCATGGGTAGTACTGGATA------GTGCTGTCGACCTCTCA
M. caribbica WM10.15 (JN183445.1)                       GTATT-------GGCATGGGTAGTACTGGATA------GTGCTGTCGACCTCTCA
M. caribbica UCLM 44A (GQ340430.1)                      GTATT-------GGCATGGGTAGTACTGGATA------GTGCTGTCGACCTCTCA
Candida fermentati ATCC 22995 (AF022718.1)             GTATT-------GGCATGGGTAGTACTGGATA------GTGCTGTCGACCTCTCA
M. caribbica S58-1 (GU943488.1)                         GTATT-------GGCATGGGTAGTACTGGATA------GTGCTGTCGACCTCTCA
M. caribbica H5MandK13 (FM199963.1)                     GTATT-------GGCATGGGTAGTACTGGATA------GTGCTGTCGACCTCTCA
M. caribbica I-5 (AB260139.1)                          GTATT-------GGCATGGGTAGTACTGGATA------GTGCTGTCGACCTCTCA
```

D

```
                                              575  580       590       600       610       620       630
                                              |....|....|....|....|....|....|....|....|....|....|....|
Clustal Consensus                             **** **************    ***     *         *** *** * *   *
Saccharomyces_cerevisiae S288c (NC_001144)    GGTAGTGAGTGATACTCTTTGGAGTTAACTTGAAATTGCTGGCCTTTTCATTGGAT
M. guilliermondii ATCC 6260T(AY939792.1)      GGTATTGAGTGATACTCTT---AGTCGGACTA--------GGCGTTTGCTTGAAAA
M. guilliermondii WM10.14 (JN183444.1)        GGTATTGAGTGATACTCTT---AGTCGGACTA--------GGCGTTTGCTTGAAAA
M. guilliermondii CanR-56 (JF817286.1)        GGTATTGAGTGATACTCTT---AGTCGGACTA--------GGCGTTTGCTTGAAAA
M. guilliermondii PX-PAT (GQ497898.1)         GGTATTGAGTGATACTCTT---AGTCGGACTA--------GGCGTTTGCTTGAAAA
M. guilliermondii SD 337 (GQ334393.1)         GGTATTGAGTGATACTCTT---AGTCGGACTA--------GGCGTTTGCTTGAAAA
M. guilliermondii EQ (EU177579.1)             GGTATTGAGTGATACTCTT---AGTCGGACTA--------GGCGTTTGCTTGAAAA
M. guilliermondii G7A1 (DQ680842.1)           GGTATTGAGTGATACTCTT---AGTCGGACTA--------GGCGTTTGCTTGAAAA
M. guilliermondii JHSd (DQ663478.1)           GGTATTGAGTGATACTCTT---AGTCGGACTA--------GGCGTTTGCTTGAAAA
M. guilliermondii L2-8 (DQ663476.1)           GGTATTGAGTGATACTCTT---AGTCGGACTA--------GGCGTTTGCTTGAAAA
M. guilliermondii UR 9406-03 (AY939795.1)     GGTATTGAGTGATACTCTT---AGTCGGACTA--------GGCGTTTGCTTGAAAA
M. guilliermondii HJM (EF191048.1)            GGTATTGAGTGATACTCTT---AGTCGGACTA--------GGCGTTTGCTTGAAAA
M. caribbica CBS 9966T(CBS Collection)        GGTATTGAGTGATACTCTT---AGTCGAACTA--------GGCGTTTGCTTGAAAA
M. caribbica IWBT-Y836 (JQ993381.1)           GGTATTGAGTGATACTCTT---AGTCGAACTA--------GGCGTTTGCTTGAAAA
M. caribbica CNRMA 200700593 (EU569001.1)     GGTATTGAGTGATACTCTT---AGTCGAACTA--------GGCGTTTGCTTGAAAA
M. caribbica IMUFRJ 51970 (FN428931.1)       GGTATTGAGTGATACTCTT---AGTCGAACTA--------GGCGTTTGCTTGAAAA
M. caribbica CBS 2022 (EU568913.1)            GGTATTGAGTGATACTCTT---AGTCGAACTA--------GGCGTTTGCTTGAAAA
M. caribbica WM10.15 (JN183445.1)             GGTATTGAGTGATACTCTT---AGTCGAACTA--------GGCGTTTGCTTGAAAA
M. caribbica UCLM 44A (GQ340430.1)            GGTATTGAGTGATACTCTT---AGTCGAACTA--------GGCGTTTGCTTGAAAA
Candida fermentati ATCC 22995 (AF022718.1)   GGTATTGAGTGATACTCTT---AGTCGAACTA--------GGCGTTTGCTTGAAAA
M. caribbica S58-1 (GU943488.1)              GGTATTGAGTGATACTCTT---AGTCGAACTA--------GGCGTTTGCTTGAAAA
M. caribbica H5MandK13 (FM199963.1)          GGTATTGAGTGATACTCTT---AGTCGAACTA--------GGCGTTTGCTTGAAAA
M. caribbica I-5 (AB260139.1)                GGTATTGAGTGATACTCTT---AGTCGAACTA--------GGCGTTTGCTTGAAAA
```

E

```
                                              321       330       340       350       360       370  375
                                              ....|....|....|....|....|....|....|....|....|....|....|
Clustal Consensus                             * * ******* **        ******      *   * * ***
Saccharomyces_cerevisiae S288c (NC_001144)    ACAAACAATTTTATCTATTCATTAAATTTTTGTCAAAAACAAGAATTTCGTAAC
M. guilliermondii ATCC 6260T(AY939792.1)      AAACACAATTTAATT----------ATTTTTACAGTTAGTCAAATTTT-------
M. guilliermondii WM10.14 (JN183444.1)        AAACACAATTTAATT----------ATTTTTACAGTTAGTCAAATTTT-------
M. guilliermondii CanR-56 (JF817286.1)        AAACACAATTTAATT----------ATTTTTACAGTTAGTCAAATTTT-------
M. guilliermondii PX-PAT (GQ497898.1)         AAACACAATTTAATT----------ATTTTTACAGTTAGTCAAATTTT-------
M. guilliermondii SD 337 (GQ334393.1)         AAACACAATTTAATT----------ATTTTTACAGTTAGTCAAATTTT-------
M. guilliermondii EQ (EU177579.1)             AAACACAATTTAATT----------ATTTTTACAGTTAGTCAAATTTT-------
M. guilliermondii G7A1 (DQ680842.1)           AAACACAATTTAATT----------ATTTTTACAGTTAGTCAAATTTT-------
M. guilliermondii JHSd (DQ663478.1)           AAACACAATTTAATT----------ATTTTTACAGTTAGTCAAATTTT-------
M. guilliermondii L2-8 (DQ663476.1)           AAACACAATTTAATT----------ATTTTTACAGTTAGTCAAATTTT-------
M. guilliermondii UR 9406-03 (AY939795.1)     AAACACAATTTAATT----------ATTTTTACAGTTAGTCAAATTTT-------
M. guilliermondii HJM (EF191048.1)            AAACACAATTTAATT----------ATTTTTACAGTTAGTCAAATTTT-------
M. caribbica CBS 9966T(CBS Collection)        AAACACAATTTAATT----------ATTTTTATTGATAGTCAAATTTT-------
M. caribbica IWBT-Y836 (JQ993381.1)           AAACACAATTTAATT----------ATTTTTATTGATAGTCAAATTTT-------
M. caribbica CNRMA 200700593 (EU569001.1)     AAACACAATTTAATT----------ATTTTTATTGATAGTCAAATTTT-------
M. caribbica IMUFRJ 51970 (FN428931.1)       AAACACAATTTAATT----------ATTTTTATTGATAGTCAAATTTT-------
M. caribbica CBS 2022 (EU568913.1)            AAACACAATTTAATT----------ATTTTTATTGATAGTCAAATTTT-------
M. caribbica WM10.15 (JN183445.1)             AAACACAATTTAATT----------ATTTTTATTGATAGTCAAATTTT-------
M. caribbica UCLM 44A (GQ340430.1)            AAACACAATTTAATT----------ATTTTTATTGACAGTCAAATTTT-------
Candida fermentati ATCC 22995 (AF022718.1)   AAACACAATTTAATT----------ATTTTTATTGATAGTCAAATTTT-------
M. caribbica S58-1 (GU943488.1)              AAACACAATTTAATT----------ATTTTTATTGATAGTCAAATTTT-------
M. caribbica H5MandK13 (FM199963.1)          AAACACAATTTAATT----------ATTTTTATTGATAGTCAAATTTT-------
M. caribbica I-5 (AB260139.1)                AAACACAATTTAATT----------ATTTTTATTGATAGTCAAATTTT-------
```

F

```
                                              565  570       580       590       600       610       620
                                              |....|....|....|....|....|....|....|....|....|....|....|
Clustal Consensus                                * * ******* **************    ***     *         *** **
Saccharomyces_cerevisiae S288c (NC_001144)    ATTCTG-TTTGGTAGTGAGTGATACTCTTTGGAGTTAACTTGAAATTGCTGGCCTT
M. guilliermondii ATCC 6260T(AY939792.1)      CCCCGGGTTTGGTATTGAGTGATACTCTT---AGTCGGACTA--------GGCGTT
M. guilliermondii WM10.14 (JN183444.1)        CCCCGGGTTTGGTATTGAGTGATACTCTT---AGTCGGACTA--------GGCGTT
M. guilliermondii CanR-56 (JF817286.1)        CCCCGGGTTTGGTATTGAGTGATACTCTT---AGTCGGACTA--------GGCGTT
M. guilliermondii PX-PAT (GQ497898.1)         CCCCGGGTTTGGTATTGAGTGATACTCTT---AGTCGGACTA--------GGCGTT
M. guilliermondii SD 337 (GQ334393.1)         CCCCGGGTTTGGTATTGAGTGATACTCTT---AGTCGGACTA--------GGCGTT
M. guilliermondii EQ (EU177579.1)             CCCCGGGTTTGGTATTGAGTGATACTCTT---AGTCGGACTA--------GGCGTT
M. guilliermondii G7A1 (DQ680842.1)           CCCCGGGTTTGGTATTGAGTGATACTCTT---AGTCGGACTA--------GGCGTT
M. guilliermondii JHSd (DQ663478.1)           CCCCGGGTTTGGTATTGAGTGATACTCTT---AGTCGGACTA--------GGCGTT
M. guilliermondii L2-8 (DQ663476.1)           CCCCGGGTTTGGTATTGAGTGATACTCTT---AGTCGGACTA--------GGCGTT
M. guilliermondii UR 9406-03 (AY939795.1)     CCCCGGGTTTGGTATTGAGTGATACTCTT---AGTCGGACTA--------GGCGTT
M. guilliermondii HJM (EF191048.1)            CCCCGGGTTTGGTATTGAGTGATACTCTT---AGTCGGACTA--------GGCGTT
M. caribbica CBS 9966T(CBS Collection)        CCCCGGGTTTGGTATTGAGTGATACTCTT---AGTCGAACTA--------GGCGTT
M. caribbica IWBT-Y836 (JQ993381.1)           CCCCGGGTTTGGTATTGAGTGATACTCTT---AGTCGAACTA--------GGCGTT
M. caribbica CNRMA 200700593 (EU569001.1)     CCCCGGGTTTGGTATTGAGTGATACTCTT---AGTCGAACTA--------GGCGTT
M. caribbica IMUFRJ 51970 (FN428931.1)       CCCCGGGTTTGGTATTGAGTGATACTCTT---AGTCGAACTA--------GGCGTT
M. caribbica CBS 2022 (EU568913.1)            CCCCGGGTTTGGTATTGAGTGATACTCTT---AGTCGAACTA--------GGCGTT
M. caribbica WM10.15 (JN183445.1)             CCCCGGGTTTGGTATTGAGTGATACTCTT---AGTCGAACTA--------GGCGTT
M. caribbica UCLM 44A (GQ340430.1)            CCCCGGGTTTGGTATTGAGTGATACTCTT---AGTCGAACTA--------GGCGTT
Candida fermentati ATCC 22995 (AF022718.1)   CCCCGGGTTTGGTATTGAGTGATACTCTT---AGTCGAACTA--------GGCGTT
M. caribbica S58-1 (GU943488.1)              CCCCGGGTTTGGTATTGAGTGATACTCTT---AGTCGAACTA--------GGCGTT
M. caribbica H5MandK13 (FM199963.1)          CCCCGGGTTTGGTATTGAGTGATACTCTT---AGTCGAACTA--------GGCGTT
M. caribbica I-5 (AB260139.1)                CCCCGGGTTTGGTATTGAGTGATACTCTT---AGTCGAACTA--------GGCGTT
```

Figure S3

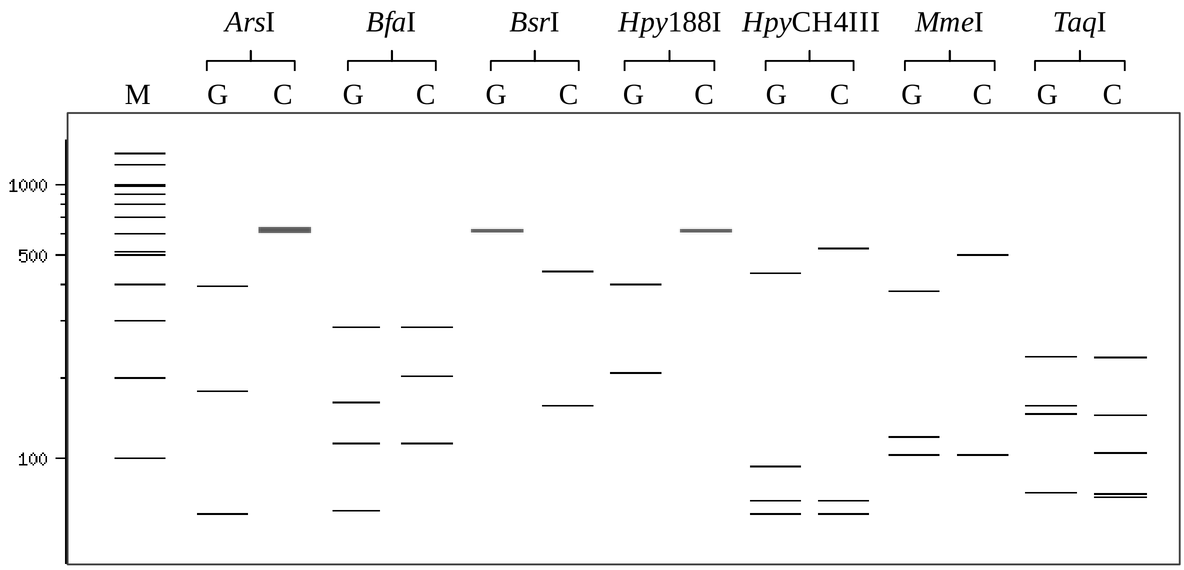
ArsI
BfaI
BsrI
Hpy188I
HpyCH4III
MmeI
TaqI
M G C G C G C G C G C G C G C
1000
500
100

Figure S4

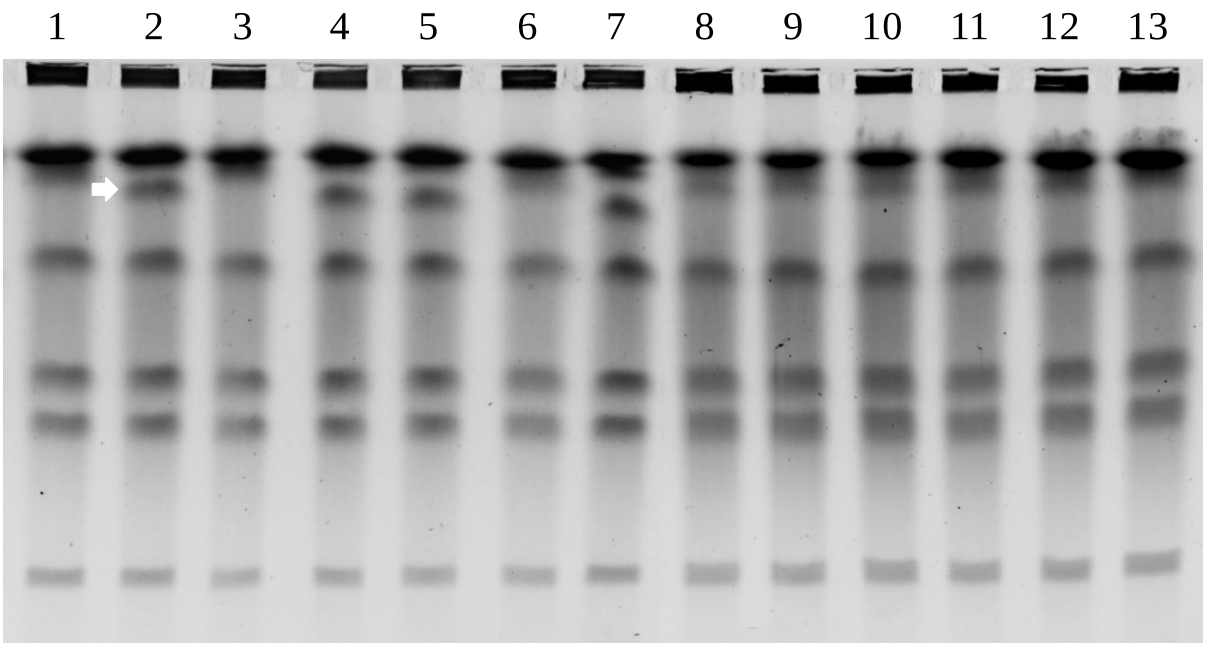

Supplement: Additional file 2: Figure S1 — Neighbour-joining phylogenetic tree based on LSU rRNA gene D1/D2 sequences showing taxa-nonspecific segregation of M. guilliermondii strains. The tree was constructed based on the evolutionary distance calculated using Kimura-2 parameter from the representative nucleotide sequences of M. guilliermondii and M. caribbica (position 13 to 308 of LSU rRNA gene of S. cerevisiae CBS 1171, GenBank Accession No. AY048154.1). The percentage of replicate trees in which the associated taxa clustered together in the bootstrap test (1000 replicates) is shown next to the branches. The bar represents 1% sequence divergence. GenBank accession numbers are mentioned within the parentheses. S. cerevisiae was the outgroup in the analysis. T = Type strain. Figure S2. In silico identified restriction enzymes which distinctly differentiated M. guilliermondii from M. caribbica. Multiple sequence alignment of representative ITS1-5.8S-ITS2 sequences of various strains of the two species obtained from NCBI GenBank and CBS yeast database showing position of identified ArsI (A), BfaI (B), BsrI (C), Hpy188I (D), HpyCH4III (E), and MmeI (F) restriction recognition sites (highlighted) which distinctly differentiated the two species. The nucleotide position was based on the sequence of the in silico PCR amplicon of ITS1-5.8S-ITS2 of S. cerevisiae strain S288c (NC_001144) including gaps generated during multiple sequence alignment. C. fermentati is the anamorph of M. caribbica. T = Type strain. Figure S3. In silico restriction digestion profile of M. guilliermondii and M. caribbica ITS1-5.8S-ITS2 amplicon. The theoretical restriction digestion profile was generated using NEBcutter, version 2.0 (http://tools.neb.com/NEBcutter2/). Lane G: M. guilliermondii ATCC 6260; Lane C: M. caribbica CBS 9966; Lane M: 100 bp DNA ladder. Figure S4. Strain level diversity of M. guilliermondii revealed by PFGE karyotyping. Lane 1 − 13: Isolates A3S2Y1, Kw1S2Y1, Kw3S3Y1, A3S6Y1, A2S6Y1, A1S9Y1, A1S9Y5, A2S9Y1, A3S9Y1, [file 1471-2180-14-52-S2.pdf]
